# Effects of endotracheal suctioning duration cerebral oxygenation in preterm infants

**DOI:** 10.1111/jocn.17382

**Published:** 2024-07-29

**Authors:** Ozlem Selime Merter, Semiha Dertli, Erdal Taskin, Mustafa Aydin, Samet Benli

**Affiliations:** ^1^ Faculty of Health Sciences Department of Nursing Firat University Elazig Turkey; ^2^ Division of Neonatology, Department of Pediatrics University of Firat Elazig Turkey; ^3^ Neonatology Department Cengiz Gokcek Gynecology and Pediatrics Hospital Gaziantep Turkey

**Keywords:** cerebral oxygenation, endotracheal aspiration duration, nurse, preterm

## Abstract

**Aim:**

This study evaluates the effects of endotracheal suctioning duration on cerebral oxygenation and physiological parameters in preterm infants in intensive care.

**Design:**

Prospective and observational study.

**Method:**

In this study, 51 preterm infants born at 28–34 weeks of gestation in NICU were evaluated. Cerebral oxygenation was measured before, during, and after endotracheal suctioning with near‐infrared spectroscopy. Pain levels of the infants were with N‐PASS scale.

**Results:**

A negative correlation was found between the lowest cerebral oxygenation value during endotracheal suctioning and the duration of endotracheal suctioning. Cerebral oxygenation levels during endotracheal suctioning were lower than pre‐ and post‐endotracheal suctioning levels. Higher cerebral oxygenation was observed in infants whose endotracheal aspiration time was less than 13 s. The duration of endotracheal suctioning was positively correlated with pain and cerebral oxygenation stabilization time.

**Conclusion:**

Prolonged endotracheal suctioning duration negatively affects cerebral oxygenation in preterm infants. The study suggests an optimal endotracheal suctioning duration under 13 s.

**Implications for the Profession and/or Patient Care:**

Properly executed endotracheal suctioning, with the correct technique and knowledge, can alleviate the adverse physiological effects observed in preterm infants and contribute to routine nursing care in intensive care units.

**Reporting Method:**

This study has been reported in line with STROBE checklists.

**Patient or Public Contribution:**

No patient or public contribution was required to design or undertake this research. Patients contributed only to the data collection.

**Impact:**

This study contributes to defining the ideal endotracheal aspiration duration, as there is not enough data so far. It showed the effect of prolonged endotracheal aspiration time on cerebral oxygenation, pain and physiological parameters in preterm infants.

What does this paper contribute to the wider global community?

- Endotracheal suctioningis a routine procedure in intensive care units aimed at clearing airways and removing secretions.
- Prolonged duration of endotracheal suctioning is associated with decreased cerebral oxygenation in preterm infants.
- There is uncertain evidence about optimal duration of endotracheal suctioning.
- Our study provides evidence for the optimal duration of endotracheal suctioning in preterm infants based on cerebral oxygenation.

## 1 INTRODUCTION

Preterm birth is one of the most challenging problems and is closely associated with mortality and morbidity. Preterm infants require neonatal intensive care (NICU) to survive due to both immaturity and various health problems (Yue et al., 2021). Respiratory problems such as apnea, respiratory distress syndrome (RDS) and asphyxia are common in preterm infants after birth (Harrison & Goldenberg, 2016). Most of these infants require ventilation until they are mature and strong enough to breathe on their own. This is usually done by intubation. There are some problems associated with intubation in preterm infants and some of these can develop due to endotracheal aspiration (ETS) (Yue et al., 2021).

ETS is a common procedure used in intensive care units to clear airways, remove secretions, and prevent atelectasis and lung infections (Cerbo et al., 2012; Diaconu et al., 2018; Gil‐Perotin et al., 2012; Morrow & Argent, 2008). Although, ETS is essential for effective breathing, it can lead to adverse effects such as hypoxia, cardiac arrhythmias, blood pressure fluctuations, and increased intracranial pressure (Chegondi et al., 2018; Schults et al., 2018). Also, it can result in secondary brain damage, neurodevelopmental problems, and mortality (Katheria et al., 2021; Schults et al., 2021). During intubation, inadequate humidification of the inspired air and the presence of an endotracheal tube can lead to airway irritation and increased secretion (Morrow & Argent, 2008). Therefore, ETS should be used to remove secretions in mechanically ventilated infants.

There is some evidence on the effective use, application and techniques of ETS (Blakeman et al., 2022; Morrow & Argent, 2008). There are recommendations and clinical guidelines in the literature regarding suction pressures, depth of suction catheter insertion and catheter size, but few of these have been objectively proven to be appropriate or safe. Existing guidelines only address the size of the cross‐sectional diameter of suction catheters and do not take into account variations in mucus properties. They also do not consider the relationship between endotracheal tube and catheter length, diameter and suction pressures. Research in clinical settings shows that practice guidelines and protocols vary widely and are generally not based on sound evidence (Morrow & Argent, 2008). However, a clinical practice guideline published in 2022 provides current clinical recommendations for neonates. According to this guideline, aspiration should not be planned in neonates, but should be performed when there is an increase in airway resistance. Suction catheters should occlude no more than 70% of the lumen of endotracheal tube in neonates and the suction pressure should be maintained below −120 mmHg. Each suction should be limited to a maximum of 15 s (Blakeman et al., 2022).

ETS in preterm infants has been reported to be one of the most common and painful procedures that can affect vital signs and other parameters (Cai et al., 2023; Zanelat et al., 2017). Exposure to pain has been associated with critical and lasting effects on brain development in preterm infants. Prolonged and repeated exposure to pain has been reported to increase physiological responses, blunt behavioural responses, alter pain thresholds and alter the development of the hypothalamic–pituitary–adrenal axis in preterm infants (Campbell‐Yeo et al., 2022).

The fact that pain is subjective and complex which makes the assessment of pain in the newborn difficult. Neonatal pain symptoms are assessed behaviourally, physiologically, hormonally or neurophysiologically (Campbell‐Yeo et al., 2022). More than 40 published neonatal pain assessment scales (PIPS, FLACC, N‐PASS, NIPS, etc.) have been reported (De Melo et al., 2014; Eriksson & Campbell‐Yeo, 2019; Olsson et al., 2021). Although the American Academy of Paediatrics advocates the use of reliable methods for accurate pain classification and management in infants (Lim & Godambe, 2017), there is still no universally accepted tool for accurate pain assessment for infants in clinical settings (Roué et al., 2018).

Near‐infrared spectroscopy (NIRS) is an emerging technique for the detection of both pain and brain damage. NIRS works by non‐invasively passing light waves through the skin and skull to measure cerebral haemodynamics in neonates and offers new possibilities in this field (Litscher et al., 1997). It allows precise identification of even small local changes in tissue oxygenation. NIRS facilitates the measurement of cerebral oxygenation (crSO2), allowing healthcare professionals to assess cerebral blood flow and perfusion pressure, and assisting in the early detection of brain damage (Jeon, 2019). NIRS is simple and can be utilized bedside in the clinic without the need for elaborate laboratory equipment (Bokiniec et al., 2012).

Although ETS is a frequent clinical procedure, there is an absence of uniform standards for various elements of the procedure (Gonçalves et al., 2015). The guidelines provided by the American Association for Respiratory Care (AARC) offer some directions for clinicians but are restricted by the lack of comprehensive evidence (Blakeman et al., 2022). Given the uncertain evidence about the appropriate duration of ETS and its correlation with potential complications, assessing the adverse effects of ETS over different durations becomes crucial. Consequently, this study focuses on evaluating the impact of ETS duration on cerebral oxygenation in preterm infants using NIRS, along with physiological and behavioural measures.

### 1.1 Hypothesis

The formulated hypotheses are:

Hypothesis 1 The longer the duration of ETS the less the crSO2 measured using NIRS in preterm infants.

Hypothesis 2 The longer the ETS impacts the greater the pain levels on the N‐PASS in preterm infants as measured on the.

Hypothesis 3 Longer ETS results in decreased heart rate and SPO2 in preterm infants.

## 2 METHODS

### 2.1 Study design

This prospective and observational study was conducted in the third level Neonatal Intensive Care Unit (NICU) of a university hospital between April and November 2022.

#### 2.1.1 Sample size

The study population comprised preterm infants receiving treatment and care in the NICU of a hospital. The study included fifty‐one preterm infants who met the selection criteria. The adequacy of the study group size was evaluated through post hoc power analysis. The post hoc power analysis was performed using the G*Power (3.1.9.7) software for the correlation between ETS duration and crSO2 during ETS with an alpha error margin of 5% (one‐way), the power was 95%, and the number of study groups was determined to be sufficient.

### 2.2 Participants

Inclusion Criteria: (1) Infants born at 28–34 weeks of gestation, (2) whose vital signs were stable for at least 24 h, (3) currently intubated and (4) whose written and verbal consents were obtained from their parents were included in the study. Exclusion criteria of infants: (1) congenital anomalies, (2) necrotizing enterocolitis, (3) asphyxia, (4) intraventricular haemorrhage, (5) sepsis, (6) sedation and analgesics, (7) high frequency ventilation requirement.

### 2.3 Data collection

‘Descriptive Information Form’ and ‘The Infant Follow‐up Form’ were used to collect data in the study. Furthermore, the pain experienced by the infants during the ETS procedure was assessed using the Neonatal Pain Agitation and Sedation Scale (N‐PASS) scale.

*Descriptive information form*: It is a form that contains demographic data about the infants in the study, such as gender, mode of birth, week of gestation, height, weight, head circumference, diagnosis, type of nutrition and day of life.

*The Infant follow‐up form*: It is the form in which the physiological findings (heart rate, SPO2 and crSO2) of infants during the ETS procedure are recorded.

### 2.4 N‐PASS (neonatal pain agitation and sedation scale)

The N‐PASS scale, developed by Hummel‐Puchalski in 2000, is designed to evaluate persistent acute pain and sedation levels in both preterm and full‐term infants (Hummel et al., 2008). The scale takes into account factors such as crying, irritability, behavioural state, facial expressions, limb tone, and vital signs. Pain is assessed using a scale ranging from 0 to 10. A score of up to four indicates mild pain, while a score of five or higher indicates moderate to severe pain. This scale is applicable to preterm infants born as early as 23 weeks of gestation up to 100 days old. The final score is adjusted by adding extra points based on the gestational age (Eroğlu & Arslan, 2018). the test–retest reliability of the N‐PASS has been demonstrated with a high Spearman's ρ correlation coefficient of .874, indicating consistent results over time. Additionally, internal consistency, as measured by Cronbach's α, is reported as very good, ranging from .84 to .89, which suggests that the items within the scale are strongly correlated with each other. Moreover, interrater reliability of the N‐PASS is deemed excellent, with Pearson's correlations ranging from .95 to .97, indicating strong agreement among different raters. These findings collectively support the robustness and reliability of the N‐PASS as a tool for assessing pain in neonates (Benbrook et al., 2023; O'Neal & Olds, 2016).

### 2.5 Cerebral oxygenation monitoring

In this process, the crSO2 levels of infants are measured using NIRS, which analyzes real‐time fluctuations in the concentrations of haemoglobin and oxyhemoglobin in tissues. NIRS presents these measurements as ‘Regional Oxygen Saturation (rSO2)’, calculating the percentage of oxyhemoglobin in relation to the total haemoglobin in the examined area. NIRS‐based oximetry has been recommended as a ‘standard of care’ and is now extensively utilized in NICUs (Cerbo et al., 2012). Typically, the standard crSO2 value for neonates is considered to be between 60% and 80%. This method enables the identification of sudden changes in cerebral blood flow and the ongoing monitoring of both cerebral and somatic oxygen levels (Jeon, 2019; Sorensen et al., 2008). In our study, the crSO2 values of the infants were assessed using the INVOS 5100C saturation monitor (Lian et al., 2020; Si et al., 2023).

### 2.6 Intervention

In the NICU, preterm infants were assessed based on set inclusion criteria. Key physiological parameters such as crSO2, pulse rate, SPO2, and pain were collected during one of the first three ETS procedures needed after mechanical ventilation was initiated post‐intubation. The NIRS device's probe was attached to the anterior frontal region to measure crSO2 in infants requiring ETS. ETS was carried out using 6 french aspiration catheter at 100 mmHg pressure for infants aged 28–34 weeks. To avoid bias, all the ETS procedures were performed by the same nurse who is very experienced in this preocedure during day shift. The entire ETS process was videotaped. Recording commenced 1 min before ETS and lasted until the infant's crSO2 levels stabilized. Routine hyperoxygenation of infants before and after ETS was not practiced. Continuous monitoring was employed to record physiological parameters before, during, and after ETS. The monitor provided heart rate and SPO2 readings, while crSO2 levels were observed directly from the NIRS device. Monitoring crSO2 levels was continued until the crSO2 levels were stabil. For crSO2 levels before and after ETS, the first measured values before and after the process were recorded, while for crSO2 levels during ETS, the lowest values measured during the process were recorded. Pain behaviours of infants were measured with the N‐PASS scale during ETS. For the N‐PASS score, the video recordings were watched and evaluated by two academicians who are experts in child health and disease nursing, and the pain scores given by the two experts were determined by taking the average. Any intervention was used to reduce pain in infants during ETS. The study recorded ETS duration and the time it took for crSO2 values to stabilize using a stopwatch based on the time in the video. The variation in crSO2 values before and during ETS, unique for each infant, was calculated as delta crSO2 (ΔcrSO2).

### 2.7 Data analysis

IBM SPSS version 22.0 was used for data analysis. The normality of the research data distribution was tested using the Kolmogorov–Smirnov test. A *p*‐value of less than .05 was considered statistically significant. Descriptive statistics, including percentages, median, and range (minimum‐maximum values), were calculated for the distribution of descriptive characteristics. Spearman's Rho was used to assess the correlation between two non‐normally distributed groups.

The cut‐off value was identified using the Receiver Operating Characteristic (ROC) curve. The Mann–Whitney U test was applied to compare independent continuous variables that were not normally distributed, while the Wilcoxon test was used for dependent continuous variables. Friedman's test was implemented to analyse variations in numerical measurements over time for the same parameter.

Typically, the standard crSO2 value for neonates is considered to be between 60% and 80% (Jeon, 2019; Sorensen et al., 2008). After data collection, infants who had a crSO2 below 60% during ETS were determined and a threshold value was defined for the duration of ETS that crSO2 fell below 60%, if a relationship was observed between the duration of ETS and crSO2. We then categorized infants into two groups based on this threshold: Group 1, with ETS duration shorter than the threshold, and Group 2, with duration longer than the threshold. We subsequently compared the physiological parameters of these two groups.

### 2.8 Ethical considerations

This study was conducted after receiving ethical clearance from the University Clinical Research Ethics Committee (approval number: 2022/05‐41) and authorization from the hospital. All healthcare professionals, including nurses and doctors in the intensive care unit, were informed about the study details. During the data collection phase, parents of eligible infants were approached, informed about the research, and their written and verbal consent was obtained.

## 3 RESULTS

**FIGURE 1:**
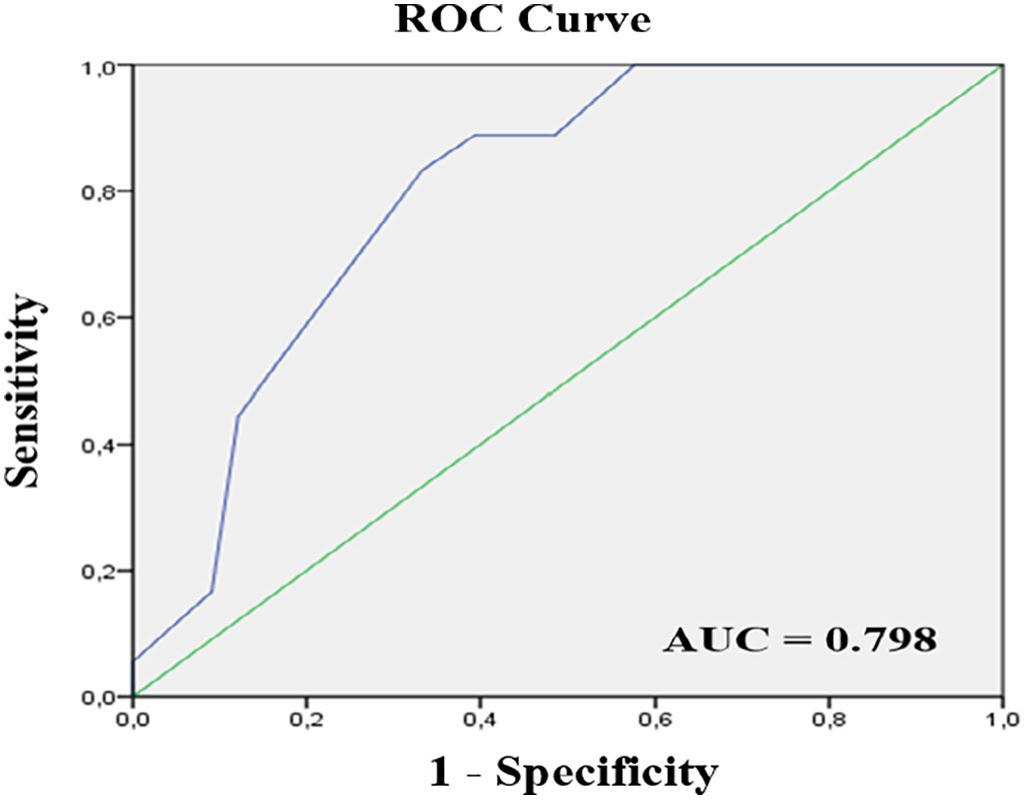
Model ROC curve analysis. [Colour figure can be viewed at wileyonlinelibrary.com]

The study included 51 preterm infants. The demographic data were shown in Table 1. The study found a negative correlation between the lowest crSO2 value during ETS and ETS duration (correlation coefficient = −.439, *p* = .01). Median ETS duration was 15 s (range: 6–19 s). An ETS duration threshold of 13 s was identified through ROC curve analysis for decreasing crSO2 below 60% during ETS, with 89% sensitivity and 61% specificity (AUC = .798, Figure 1). Infants were then categorized into two groups: Group 1 (ETS duration ≤13 s, *n* = 22) and Group 2 (ETS duration >13 s, n = 29). Median ETS durations were 10 s (min: 6, max: 13) and 16 s (min: 14, max: 16) for Group 1 and Group 2, respectively. The day of life was similar between groups (*p* = .858). The physiological parameters of both groups are compared in Table 2. There were no significant differences in heart rate between the groups before, during, or after ETS (*p* = .206, *p* = .371 and *p* = .332, respectively). Group 1 had higher SPO2 values before and during ETS than Group 2 (*p* = .041 and *p* = .009, respectively), but post‐ETS values were similar (*p* = .075). CrSO2 values were comparable before and after ETS (*p* = .864 and *p* = .44, respectively); however, Group 1 had significantly higher crSO2 than Group 2 (*p* = .009). Group 2 exhibited significantly higher ΔcrSO2 values than Group 1 (*p* < .001). Additionally, Group 2 had notably longer crSO2 recovery times (*p* < .001) and significantly higher N‐PASS scores compared to Group 1 (*p* = .022).

**TABLE 1:** Descriptive characteristics of preterm infants.

Descriptive characteristics	*n*	%
Gender		
Female	25	49
Male	26	51
Mode of delivery		
Vaginal	2	4
Caesarean section	49	96
Diagnosis		
PM	26	51
PM + RDS	25	49
Type of nutrition		
Breast milk	30	59
TPN	18	35
Infant Formula	3	6
	x̄ ± SD	Med (Min–Max)
Week of gestation	31.56 ± 2.31	32 (28–34)
Height (cm)	40.29 ± 3.99	41 (31–48)
Weight (gr)	1534.45 ± 583.90	1460 (590–2970)
Head circumference (cm)	29.03 ± 3.26	30 (23–35)
Day of life	6.96 ± 4.45	5 (2–20)

Abbreviations: PM, prematurity; RDS, respiratory distress syndrome; TPN, total parenteral nutrition.

**TABLE 2:** Comparison of physiological parameters before, during and after ETS according to groups.

Physiological parameters	Group 1 (*n* = 22)		Group 2 (*n* = 29)		* *p*‐value
	Med	Min–max	Med	Min–max	
Heart rate					
Before ETS	139.5	120–154	144	108–175	.206
During ETS	136.5	95–157	125	92–171	.371
After ETS	139	126–158	142	82–164	.332
SPO2					
Before ETS	94.5	90–96	93	88–96	**.041**
During ETS	90	77–94	87	50–94	**.009**
After ETS	96	91–96	95	73–96	.075
crSO2					
Before ETS	70	57–91	70	59–91	.864
During ETS	65.5	51–82	57	43–81	**.009**
After ETS	70	54–94	68	59–94	.440
crSO2 stabilization time (sec)	95	50–310	250	55–360	**.000**
ΔcrSO2	4	1–17	10	1–27	**.000**
N‐PASS	7	4–10	8	3–10	**.022**

* Mann Whitney *U* test.

Bold values denote statistical significance at the p < 0.05 level.

Comparing crSO2 levels before, during and after ETS, it was found that crSO2 levels before and after ETS were comparable (*p* = .510). However, crSO2 levels during ETS were significantly lower than those recorded both before and after the procedure (*p* < .001 in both cases). Table 3 contains data comparing pulse rate and SPO2 before, during, and after ETS.

**TABLE 3:** Comparison of physiological parameters before, during and after ETS for entire group (*n* = 51).

Physiological parameters		Before vs during ETS	During vs after ETS	Before vs after ETS
Heart rate	Median (min‐max)	142 (108–175) vs 135 (38–171)	135 (38–171) vs 140 (82–164)	142 (108–175) vs 140 (82–164)
	*p*‐value*	**.045**	.087	.761
SPO2	Median(min‐max)	94 (88–96) vs 89 (50–94)	89 (50–94) vs 95 (73–96)	94 (88–96) vs 95 (73–96)
	*p*‐value*	**<.001**	**<.001**	**.013**
crSO2	Median(min‐max)	70 (57–91) vs 63 (43–82)	63 (43–82) vs 69 (54–94)	70 (57–91) vs 69 (54–94)
	*p*‐value*	**<.001**	**<.001**	.510

* Wilcoxon Test.

Bold values denote statistical significance at the p < 0.05 level.

This study found positive correlations between the duration of ETS and the N‐PASS score (*r* = .346, *p* = .013), the time taken for crSO2 stabilization (*r* = .550, *p* < .001), ΔcrSO2 (*r* = .472, *p* < .001). Additionally, a negative correlation was observed between ETS duration and SPO2 during the procedure (*p* = .01, *r* = −.359). In addition, there was a positive correlation between ΔcrSO2 and both N‐PASS (*r* = .380, *p* < .006) and the time required for crSO2 stabilization (*r* = .678, *p* < .001). Moreover, the time for crSO2 stabilization was also positively correlated with the N‐PASS score (*r* = .425, *p* = .002), as shown in Table 4.

**TABLE 4:** Correlation of ETS duration and other variables.

Variables		N‐PASS	crSO2 stabilization time	ΔcrSO2	SPO2 during ETS
ETS Duration	*r* ^a^	.346	.550	.472	−.359
	*p*	.013	<.001	<.001	.01
ΔcrSO2	*r*	.380	.678	NA	−.232
	*p*	<.006	<.001		.102
N‐PASS	*r*	NA	.425	.380	−.469
	*p*		.002	.006	.001

^**a**^ Spearman Correlation Test.

## 4 DISCUSSION

ETS is a routine procedure in NICU for intubated infants. It is imperative to consider potential complications that may arise during this procedure (Misirlioglu et al., 2021). Some studies have noted changes in the hemodynamic parameters of patients during ETS (Maggiore et al., 2002; Misirlioglu et al., 2021; Morrow & Argent, 2008). Although guidelines recommend that the duration of ETS should ideally be less than 15 s, but the level of evidence supporting this duration is relatively limited (Gardner & Shirland, 2009; American Association of Respiratory Care (AARC), 2010; Maraş et al., 2017). Moreover, this recommendation is predominantly based on data from adult patients, with insufficient data available for newborns. Consequently, there is a lack of consensus among studies regarding the optimal ETS duration. Some studies have demonstrated that ETS can lead to a reduction in cerebral regional oxygen saturation (Mosca et al., 1997; Skov et al., 1992). Prolonged ETS poses an increased risk of tracheal mucosa damage and hypoxia (Czarnik et al., 1991; Gonçalves et al., 2015; Pedersen et al., 2009). Hypoxia is closely associated with persistent motor, sensory, and cognitive impairments, with cerebral hypoxia playing a pivotal role in this context (Millar et al., 2017). This study observed that prolonged ETS resulted in decreased crSO2, a valuable indicator of cerebral oxygenation. Utilizing a ROC analysis with a lower threshold of 60% for crSO2, the cut‐off value for ETS duration was determined to be 13 s. Based on this time threshold, infants exceeding 13 s of ETS experienced greater crSO2 reductions, and it took longer to stabilize their crSO2 levels. To the best of our knowledge, our study is the first to elucidate the relationship between ETS duration and crSO2, offering evidence for an ideal ETS duration.

In a study that examined the effect of ETS on regional oxygen saturation (rSO2) independently of its duration, 20 intubated paediatric patients in an intensive care unit were investigated. In this study, crSO2 values remained similar before, during, and after ETS, while somatic/mesenteric rSO2 values decreased during ETS compared to before. Also, pulse rate increased during ETS compared to pre‐ETS values, while SPO2 remained similar in this study (Misirlioglu et al., 2021). In an another study, Kohlhauser et al. compared rSO2, pulse rate, and SPO2 values before and during ETS between different ventilator modes. In this investigation involving 26 preterm infants, both ventilator groups exhibited lower rSO2, pulse rate, and SPO2 values during ETS than before the procedure (Kohlhauser et al., 2000). In our study, we observed lower crSO2 values during ETS compared to before and after the procedure. This discrepancy in findings may be attributed to the larger sample size in our study and the fact that our sample consisted of preterm infants. Similarly, in our study, we observed a reduction in pulse rate and SPO2 values during ETS compared to pre‐ETS levels, consistent with the findings of Kohlhauser et al.

Moreover, our study identified a positive correlation between N‐PASS pain scores and both ETS duration and ΔcrSO2. Furthermore, N‐PASS scores were higher in Group 2. In a study by Misirlioglu et al., pain scores were higher during the procedure compared to pre‐procedure scores, but no investigation into the relationship between crSO2 and pain was undertaken (Misirlioglu et al., 2021). In a recent prospective study involving 64 preterm infants, ΔcrSO2 was calculated before and during heel lance and venepuncture procedures. Pain assessment during these procedures utilized NIPS and PIPP‐R pain scores. This study revealed robust correlations between ΔcrSO2 and both NIPS and PIPP‐R scores (Kumar et al., 2023), aligning with the results of our study. Additionally, we observed a positive correlation between the time required for crSO2 normalization and N‐PASS pain scores in our investigation.

A stable crSO2 value serves as an indirect indicator of consistent cerebral blood flow (Garvey et al., 2018). Our study indicated that crSO2 stabilization took longer in Group 2. Upon reviewing the literature, no studies were found that examined the relationship between prolonged ETS and crSO2 stabilization.

### 4.1 Limitations

This study was conducted at a single center, and the assessment of ETS duration focused solely on crSO2, without considering the oxygenation of other tissues. Also, the infants had different diagnoses that could impact cerebral oxygenation.

### 4.2 Practices

Cerebral oxygenation refers to the oxygen content within the brain's arteries, veins, and capillaries (Elser et al., 2011). Since cerebral blood flow is responsible for delivering oxygen to the brain, monitoring cerebral oxygenation provides paediatric nurses and healthcare professionals with a way to observe changes in cerebral blood flow. It is crucial to ensure that intensive care unit nurses possess the necessary knowledge and skills, based on robust scientific evidence, to perform ETS and related procedures. Improperly conducted ETS can harm patients. Therefore, it is essential that nurses are well‐informed and competent in this area (Mwakanyanga et al., 2018). Properly executed ETS, with the correct technique and knowledge, can alleviate the adverse physiological effects observed in preterm infants and contribute to routine nursing care in intensive care units. In nursing practices, prioritizing patient comfort and pain management is of utmost importance. As integral members of the healthcare team, nurses spend extended periods with patients, closely monitoring their condition and assuming responsibility for pain assessment and alleviation (Düzkaya & Kuğuoğlu, 2015).

## 5 CONCLUSION

In preterm infants intubated in the NICU, accurate application of ETS and management of potential procedural complications are of great importance. The results of this study showed that the ideal aspiration time in preterm infants should not exceed 13 s. To our knowledge, this study is the first attempt to provide evidence for the optimal duration of ETS based on cerebral oxygenation. Future research may be recommended to investigate other parameters in addition to crSO2 to establish evidence‐based guidelines for determining the optimal duration of ETS. It is important for NICU nurses to focus on maintaining tissue oxygenation during ETS in preterm infants.

## AUTHOR CONTRIBUTIONS

Özlem Selime Merter contributed to the conceptualization, methodology, software usage, data curation, and the original draft preparation. Semiha Dertli was responsible for visualization, investigation, and supervision. Erdal Taskin handled software usage, validation, and contributed to writing, reviewing, and editing the manuscript. Mustafa Aydin was responsible for visualization, investigation, and supervision. Samet Benli was responsible for visualization, investigation, and supervision.

## FUNDING INFORMATION

This research did not receive any specific grant from funding agencies in the public, commercial, or not‐for‐profit sectors.

## CONFLICT OF INTEREST STATEMENT

The authors declare that they have no conflict of interests. (This manuscript has not been published anywhere and has not been submitted for publication).

## Supporting information


Data S1.

## Data Availability

The data that support the findings of this study are available on request from the corresponding author. The data are not publicly available due to privacy or ethical restrictions.

## References

[jocn17382-bib-0001] American Association of Respiratory Care (AARC) . (2010). AARC clinical practice guidelines: Endotracheal suctioning of mechanically ventilated adults and children with artificial airways. Respiratory Care, 55(6), 758–764. 20507660

[jocn17382-bib-0002] Benbrook, K. , Manworren, R. C. , Zuravel, R. , Entler, A. , Riendeau, K. , Myler, C. , & Ricca, P. (2023). Agreement of the neonatal pain, agitation, and sedation scale (N‐PASS) with NICU nurses' assessments. Advances in Neonatal Care, 23(2), 173–181. 10.1097/ANC.0000000000000968 35362716

[jocn17382-bib-0003] Blakeman, T. C. , Scott, J. B. , Yoder, M. A. , Capellari, E. , & Strickland, S. L. (2022). AARC clinical practice guidelines: Artificial airway suctioning. Respiratory Care, 67(2), 258–271. 10.4187/respcare.09548 35078900

[jocn17382-bib-0004] Bokiniec, R. , Zbiec, A. , Seliga, J. , Sawosz, P. , Liebert, A. , Klosinska, I. , Bargiel, A. , Maniewski, R. , & Kornacka, M. (2012). Assessment of brain oxygenation in term and preterm neonates using near infrared spectroscopy. Advances in Medical Sciences, 57(2), 348–355. 10.2478/v10039-012-0050-6 23159869

[jocn17382-bib-0006] Cai, Q. , Luo, W. , Zhou, Y. , Yin, Y. , Zhu, K. , Shi, H. , & Liao, Y. (2023). Efficacy and safety of non‐pharmacological interventions for endotracheal suctioning pain in preterm infants: A systematic review. Nursing Open, 10(2), 424–434. 10.1002/nop2.1364 36100551 PMC9834158

[jocn17382-bib-0007] Campbell‐Yeo, M. , Eriksson, M. , & Benoit, B. (2022). Assessment and management of pain in preterm infants: A practice update. Children, 9(2), 244. 10.3390/children9020244 35204964 PMC8869922

[jocn17382-bib-0008] Cerbo, R. , Cabano, R. , Di Comite, A. , Longo, S. , Maragliano, R. , & Stronati, M. (2012). Cerebral and somatic rSO2 in sick preterm infants. The Journal of Maternal‐Fetal Neonatal Medicine, 25(sup4), 89–92. 10.3109/14767058.2012.715030 22958033

[jocn17382-bib-0009] Chegondi, M. , Francis, T. , Lin, W.‐C. , Naqvi, S. , Raszynski, A. , & Totapally, B. R. (2018). Effects of closed endotracheal suctioning on systemic and cerebral oxygenation and hemodynamics in children. Pediatric Critical Care Medicine, 19(1), e23–e30. 10.1097/PCC.0000000000001377 29189639

[jocn17382-bib-0010] Czarnik, R. , Stone, K. , Everhart, C., Jr. , & Preusser, B. (1991). Differential effects of continuous versus intermittent suction on tracheal tissue. Heart Lung: The Journal of Critical Care, 20(2), 144–151. 2004925

[jocn17382-bib-0011] Diaconu, O. , Siriopol, I. , Poloșanu, L. I. , & Grigoraș, I. (2018). Endotracheal tube biofilm and its impact on the pathogenesis of ventilator‐associated pneumonia. The Journal of Critical Care Medicine, 4(2), 50–55. 10.2478/jccm-2018-0011 30581995 PMC6294989

[jocn17382-bib-0012] Düzkaya, D. S. , & Kuğuoğlu, S. (2015). Assessment of pain during endotracheal suction in the pediatric intensive care unit. Pain Management Nursing, 16(1), 11–19. 10.1016/j.pmn.2014.02.003 24957815

[jocn17382-bib-0013] Elser, H. E. , Holditch‐Davis, D. , & Brandon, D. H. (2011). Cerebral oxygenation monitoring: A strategy to detect intraventricular hemorrhage and periventricular leukomalacia. Newborn and Infant Nursing Reviews, 11(3), 153–159. 10.1053/j.nainr.2011.07.007 21909236 PMC3168549

[jocn17382-bib-0014] Eriksson, M. , & Campbell‐Yeo, M. (2019). Assessment of pain in newborn infants. In Seminars in fetal and neonatal medicine (Vol. 24, 101003). WB Saunders. 30987943 10.1016/j.siny.2019.04.003

[jocn17382-bib-0015] Eroğlu, A. , & Arslan, S. (2018). Perception, evaluation and management of pain in the newborn. Journal of Duzce University Health Sciences Institute, 8(1), 52–60.

[jocn17382-bib-0016] Gardner, D. L. , & Shirland, L. (2009). Evidence‐based guideline for suctioning the intubated neonate and infant. Neonatal Network, 28(5), 281–302. 10.1891/0730-0832.28.5.281 19720593

[jocn17382-bib-0017] Garvey, A. A. , Kooi, E. M. , Smith, A. , & Dempsey, E. M. (2018). Interpretation of cerebral oxygenation changes in the preterm infant. Children, 5(7), 94. 10.3390/children5070094 29987227 PMC6069134

[jocn17382-bib-0018] Gil‐Perotin, S. , Ramirez, P. , Marti, V. , Sahuquillo, J. M. , Gonzalez, E. , Calleja, I. , Menendez, R. , & Bonastre, J. (2012). Implications of endotracheal tube biofilm in ventilator‐associated pneumonia response: A state of concept. Critical Care, 16(3), 1–9. 10.1186/cc11357 PMC3580639 22621676

[jocn17382-bib-0019] Gonçalves, R. L. , Tsuzuki, L. M. , & Carvalho, M. G. S. (2015). Endotracheal suctioning in intubated newborns: An integrative literature review. Revista Brasileira de Terapia Intensiva, 27, 284–292. 10.5935/0103-507X.20150048 26465249 PMC4592124

[jocn17382-bib-0020] Harrison, M. S. , & Goldenberg, R. L. (2016, April). Global burden of prematurity. In Seminars in fetal and neonatal medicine (Vol. 21, pp. 74–79). WB Saunders. 26740166 10.1016/j.siny.2015.12.007

[jocn17382-bib-0021] Hummel, P. , Puchalski, M. , Creech, S. , & Weiss, M. (2008). Clinical reliability and validity of the N‐PASS: Neonatal pain, agitation and sedation scale with prolonged pain. Journal of Perinatology, 28(1), 55–60. 18165830 10.1038/sj.jp.7211861

[jocn17382-bib-0022] Jeon, G. W. (2019). Clinical application of near‐infrared spectroscopy in neonates. Neonatal Medicine, 26(3), 121–127. 10.5385/nm.2019.26.3.121

[jocn17382-bib-0023] Katheria, A. C. , Stout, J. , Morales, A. L. , Poeltler, D. , Rich, W. D. , Steen, J. , Nuzzo, S. , & Finer, N. (2021). Association between early cerebral oxygenation and neurodevelopmental impairment or death in premature infants. Journal of Perinatology, 41(4), 743–748. 10.1038/s41372-021-00942-w 33589727 PMC7883949

[jocn17382-bib-0024] Kohlhauser, C. , Bernert, G. , Hermon, M. , Popow, C. , Seidl, R. , & Pollak, A. (2000). Effects of endotracheal suctioning in high‐frequency oscillatory and conventionally ventilated low birth weight neonates on cerebral hemodynamics observed by near infrared spectroscopy (NIRS). Pediatric Pulmonology, 29(4), 270–275. 10.1002/(SICI)1099-0496(200004)29:4<270::AID-PPUL6>3.0.CO;2-Q 10738014

[jocn17382-bib-0025] Kumar, S. , Priyadarshi, M. , Singh, P. , Pallapothu, B. , Chaurasia, S. , & Basu, S. (2023). Correlation of clinical pain scores with cerebral oxygenation in preterm neonates during acute painful procedures: A prospective observational study. Journal of Perinatology, 43(5), 584–589. 10.1038/s41372-022-01543-x 36271296

[jocn17382-bib-0026] Lian, C. , Li, P. , Wang, N. , Lu, Y. , & Shangguan, W. (2020). Comparison of basic regional cerebral oxygen saturation values in patients of different ages: A pilot study. Journal of International Medical Research, 48(8), 0300060520936868. 32833525 10.1177/0300060520936868 PMC7448148

[jocn17382-bib-0027] Lim, Y. , & Godambe, S. (2017). Prevention and management of procedural pain in the neonate: An update, American Academy of Pediatrics, 2016. Archives of Disease in Childhood‐Education, 102, 254–256. 10.1136/archdischild-2016-311066 28724533

[jocn17382-bib-0028] Litscher, G. , Schwarz, G. , Ratzenhofer‐Komenda, B. , Kovac, H. , Gabor, S. , & Smolle‐Jüttner, F. (1997). Transcranial cerebral oximetry in the hyperbaric environment‐Transkranielle zerebrale Oximetrie unter hyperbaren Bedingungen. Biomedical Engineering, 42, 38–41. 10.1515/bmte.1997.42.3.38 9112799

[jocn17382-bib-0029] Maggiore, S. M. , Iacobone, E. , Zito, G. , Conti, C. , Antonelli, M. , & Proietti, R. (2002). Closed versus open suctioning techniques. Minerva Anestesiologica, 68(5), 360–364. 12029246

[jocn17382-bib-0030] Maraş, G. B. , Güler, E. K. , Eşer, İ. , & Köse, Ş. (2017). Knowledge and practice of intensive care nurses for endotracheal suctioning in a teaching hospital in western Turkey. Intensive & Critical Care Nursing, 39, 45–54. 10.1016/j.iccn.2016.08.006 27876409

[jocn17382-bib-0031] Melo, G. M. D. , Lélis, A. L. P. D. A. , Moura, A. F. D. , Cardoso, M. V. L. M. L. , & Silva, V. M. D. (2014). Pain assessment scales in newborns: Integrative review. Revista Paulista de Pediatria, 32, 395–402. 10.1590/S0103-05822014000400017 25511005 PMC4311795

[jocn17382-bib-0032] Millar, L. J. , Shi, L. , Hoerder‐Suabedissen, A. , & Molnár, Z. (2017). Neonatal hypoxia ischaemia: Mechanisms, models, and therapeutic challenges. Frontiers in Cellular Neuroscience, 11, 78. 10.3389/fncel.2017.00078 28533743 PMC5420571

[jocn17382-bib-0033] Misirlioglu, M. , Horoz, O. O. , Yildizdas, D. , Ekinci, F. , Yontem, A. , Menemencioglu, A. , & Salva, G. (2021). The effects of endotracheal suctioning on hemodynamic parameters and tissue oxygenation in pediatric intensive care unit. Journal of Pediatric Intensive Care, 11(4), 349–354. 10.1055/s-0040-1721725 36388067 PMC9649295

[jocn17382-bib-0034] Morrow, B. M. , & Argent, A. C. (2008). A comprehensive review of pediatric endotracheal suctioning: Effects, indications, and clinical practice. Pediatric Critical Care Medicine, 9(5), 465–477. 10.1097/PCC.0b013e31818499cc 18679146

[jocn17382-bib-0035] Mosca, F. A. , Colnaghi, M. , Lattanzio, M. , Bray, M. , Pugliese, S. , & Fumagalli, M. (1997). Closed versus open endotracheal suctioning in preterm infants: Effects on cerebral oxygenation and blood volume. Biology of the Neonate, 72(1), 9–14. 10.1159/000244460 9313829

[jocn17382-bib-0036] Mwakanyanga, E. T. , Masika, G. M. , & Tarimo, E. A. (2018). Intensive care nurses' knowledge and practice on endotracheal suctioning of the intubated patient: A quantitative cross‐sectional observational study. PLoS One, 13(8), e0201743. 10.1371/journal.pone.0201743 30114257 PMC6095500

[jocn17382-bib-0037] Olsson, E. , Ahl, H. , Bengtsson, K. , Vejayaram, D. N. , Norman, E. , Bruschettini, M. , & Eriksson, M. (2021). The use and reporting of neonatal pain scales: A systematic review of randomized trials. Pain, 162(2), 353–360. 10.1097/j.pain.0000000000002046 32826760 PMC7808360

[jocn17382-bib-0038] O'Neal, K. , & Olds, D. (2016). Differences in pediatric pain management by unit types. Journal of Nursing Scholarship, 48(4), 378–386. 10.1111/jnu.12222 27275945

[jocn17382-bib-0039] Pedersen, C. M. , Rosendahl‐Nielsen, M. , Hjermind, J. , & Egerod, I. (2009). Endotracheal suctioning of the adult intubated patient—What is the evidence? Intensive & Critical Care Nursing, 25(1), 21–30. 10.1016/j.iccn.2008.05.004 18632271

[jocn17382-bib-0040] Roué, J.‐M. , Rioualen, S. , Gendras, J. , Misery, L. , Gouillou, M. , & Sizun, J. (2018). Multi‐modal pain assessment: Are near‐infrared spectroscopy, skin conductance, salivary cortisol, physiologic parameters, and neonatal facial coding system interrelated during venepuncture in healthy, term neonates? Journal of Pain Research, 11, 2257–2267. 10.2147/JPR.S165810 30349352 PMC6188070

[jocn17382-bib-0041] Schults, J. , Mitchell, M. L. , Cooke, M. , & Schibler, A. (2018). Efficacy and safety of normal saline instillation and paediatric endotracheal suction: An integrative review. Australian Critical Care, 31(1), 3–9. 10.1016/j.aucc.2017.02.069 28347624

[jocn17382-bib-0042] Schults, J. A. , Mitchell, M. L. , Cooke, M. , Long, D. A. , Ferguson, A. , & Morrow, B. (2021). Endotracheal suction interventions in mechanically ventilated children: An integrative review to inform evidence‐based practice. Australian Critical Care, 34(1), 92–102. 10.1016/j.aucc.2020.05.003 32763068

[jocn17382-bib-0043] Si, J. , Li, M. , Zhang, X. , Han, R. , Ji, X. , & Jiang, T. (2023). Cerebral tissue oximeter suitable for real‐time regional oxygen saturation monitoring in multiple clinical settings. Cognitive Neurodynamics, 17(3), 563–574. 10.1007/s11571-022-09847-6 37265661 PMC10229493

[jocn17382-bib-0044] Skov, L. , Ryding, J. , Pryds, O. , & Greisen, G. (1992). Changes in cerebral oxygenation and cerebral blood volume during endotracheal suctioning in ventilated neonates. Acta Paediatrica, 81(5), 389–393. 10.1111/j.1651-2227.1992.tb12255.x 1498503

[jocn17382-bib-0045] Sorensen, L. C. , Leung, T. S. , & Greisen, G. (2008). Comparison of cerebral oxygen saturation in premature infants by near‐infrared spatially resolved spectroscopy: Observations on probe‐dependent bias. Journal of Biomedical Optics, 13(6), 064013‐064013‐064014. 19123660 10.1117/1.3013454

[jocn17382-bib-0048] Yue, G. , Wang, J. , Li, H. , Li, B. , & Ju, R. (2021). Risk factors of mechanical ventilation in premature infants during hospitalization. Therapeutics and Clinical Risk Management, 17, 777–787. 10.2147/TCRM.S318272 34354359 PMC8331080

[jocn17382-bib-0049] Zanelat, C. F. , Rocha, F. R. , Lopes, G. M. , Ferreira, J. R. , Gabriel, L. S. , & Oliveira, T. G. (2017). The respiratory physiotherapy causes pain in newborns? A systematic review. Fisioterapia Em Movimento, 30, 177–186. 10.1590/1980-5918.030.001.AR01

